# Laparoscopic salvage in a storm: A multidisciplinary case report of thyroid storm complicated by gastrointestinal perforation

**DOI:** 10.1097/MD.0000000000043720

**Published:** 2025-08-01

**Authors:** Ling Ye, Weiqiao Zhang

**Affiliations:** a Department of Anesthesiology, The First Affiliated Hospital of Zhejiang Chinese Medical University (Zhejiang Provincial Hospital of Chinese Medicine), Hangzhou, Zhejiang, China.

**Keywords:** Burch–Wartofsky Point Scale, gastrointestinal perforation, laparoscopic repair, multidisciplinary management, thyroid storm

## Abstract

**Rationale:**

Thyroid storm (TS) is a critical endocrine emergency characterized by multiorgan failure. Although rare, gastrointestinal perforation is a catastrophic complication that frequently leads to diagnostic delays and poor prognosis. This case provides valuable clinical insights based on a multidisciplinary strategy and well-considered surgical timing.

**Patient concerns:**

A 22-year-old male with poorly controlled hyperthyroidism presented with acute abdominal pain, fever, and altered mental status.

**Diagnoses:**

He was diagnosed with TS based on clinical scoring (Burch-Wartofsky Point Scale) and laboratory markers. Imaging confirmed gastrointestinal perforation.

**Interventions:**

A multidisciplinary team initiated immediate medical stabilization using beta-blockers, antithyroid drugs, corticosteroids, and supportive care. Definitive treatment with laparoscopic repair was performed after clinical optimization under tailored anesthesia.

**Outcomes:**

The patient achieved complete recovery and was discharged on postoperative day 10, with normalization of thyroid and inflammatory markers.

**Lessons:**

This case emphasizes the importance of suspecting TS in acute abdomen cases with hyperthyroid history; that cautious surgical delay may be justified to optimize outcomes in TS; and that minimally invasive surgery combined with individualized anesthesia is feasible in this high-risk population.

## 1. Introduction

Thyroid storm (TS), an endocrine emergency characterized by multi-organ failure resulting from severe thyrotoxicosis, necessitates urgent treatment.^[1]^ Common precipitating factors include trauma, infection, illness, or surgical stress. TS manifests as an abrupt exacerbation of preexisting hyperthyroidism, with hallmark features of extreme hypermetabolism and adrenergic hyperactivity, including fever with diaphoresis, tachycardia, vomiting/diarrhea, and neurological disturbances.^[2]^ Gastrointestinal perforation represents a rare yet life-threatening complication of TS. A systematic review of literature from 2000 to 2023 identified only 11 documented global cases of TS-associated perforation, with surgical mortality exceeding 60%,^[3–6]^ highlighting the extreme perioperative mortality risk in these patients. Timely diagnosis and effective intervention within constrained timeframes are thus critical for prognosis.

The Burch–Wartofsky Point Scale (BWPS),^[7]^ proposed in 1993, has been widely applied for TS diagnosis over the past 3 decades, albeit criticized for oversensitivity and high false-positive rates. For confirmed TS patients with gastrointestinal perforation, no consensus exists regarding optimal preoperative pharmacotherapy for rapid symptom control, ideal surgical timing post-onset, or perioperative management strategies. This case report provides critical clinical insights through its multidisciplinary collaborative approach: challenging the guideline-mandated 6 to 12-hour window for emergency perforation repair, demonstrating the feasibility of laparoscopic surgery combined with TS-tailored anesthesia.

## 2. Case description

### 2.1. Chief complaints

A 22-year-old male (height: 176 cm, weight: 55 kg) presented to the emergency department with a 2-day history of persistent epigastric pain and 1 episode of tarry stool. He denied any known history of other endocrine disorders and had no relevant family history of thyroid disease. He had been diagnosed with hyperthyroidism 2 years earlier but demonstrated poor compliance with treatment. Initial treatment with omeprazole failed to alleviate symptoms, and the pain progressed to severe cramping. Vital signs on admission included tachycardia (heart rate: 120 beats/min) and normotension (blood pressure: 108/53 mm Hg). Laboratory findings revealed leukocytosis (WBC: 8.9 × 10⁹/L, neutrophils: 79.3%) and anemia (hemoglobin: 119 g/L). An abdominal computed tomography (CT) scan was initially unremarkable. A provisional diagnosis of acute diffuse peritonitis with concurrent hyperthyroidism was established.

### 2.2 Investigation and diagnostic evaluation

Following symptomatic management with phloroglucinol, esomeprazole, and ceftriaxone, partial resolution of symptoms occurred. However, clinical deterioration occurred at 20:00, marked by fever (39.1°C) and worsening tachycardia (heart rate: 145 beats/min). We immediately performed a BWPS score on the patient, which is displayed in Table 1. Given the patient’s hyperthyroidism history and a BWPS score of 65, TS was suspected. Thyroid function tests confirmed markedly elevated free triiodothyronine (FT3: 28.58 pmol/L) and free thyroxine (FT4: 44.2 pmol/L), suppressed thyroid-stimulating hormone (TSH: <0.01 mIU/L), and elevated anti-thyroglobulin (149.46 IU/mL) and antithyroid peroxidase (>1000.00 IU/mL) antibodies.

**Table 1:** Diagnostic parameters and scoring system of the Burch–Wartofsky Point Scale for thyroid storm assessment.

Diagnostic parameter	Score	Patient score
Thermoregulatory dysfunction		
Temperature (°C)		
37.2–37.7	5	
37.8–38.2	10	
38.3–38.8	15	
38.9–39.4	20	20
39.5–39.9	25	
≥40.0	30	
Cardiovascular system abnormalities		
Tachycardia (beats/min)		
100–109	5	
110–119	10	
120–129	15	
130–139	20	
≥140	25	25
Atrial fibrillation		
Absent	0	
Present	10	
Congestive heart failure		
Absent	0	
Present	10	
Congestive heart failure		
Absent	0	
Mild (pedal edema)	5	
Moderate (bibasilar rales)	10	
Severe (pulmonary edema)	20	
Gastrointestinal/hepatic dysfunction		
Absent	0	
Moderate (diarrhea, abdominal pain, nausea/vomiting)	10	10
Severe (unexplained jaundice)	15	
Central nervous system symptoms		
Absent	0	
Mild (agitation)	10	
Moderate (delirium, confusion, extreme lethargy)	20	
Severe (seizures, coma)	30	
Precipitating factors		
Absent	0	
Present	10	10
Total score interpretation		
>45: TS		65
25–45: Impending storm		
<25: Not indicative of TS		

A score of 45 or higher is highly suggestive of a TS. The patient achieved a Burch–Wartofsky Point Scale of 65 at 8:00 pm on the day of admission.

TS = thyroid storm.

**Figure 1.**
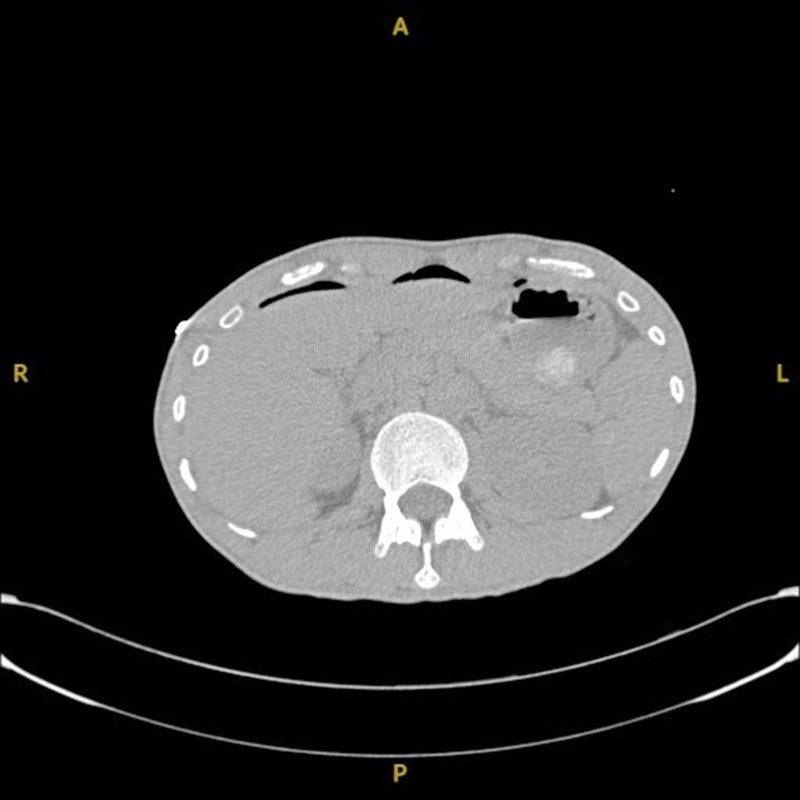
Computed tomography imaging of gastric perforation. Axial computed tomography scan demonstrating anatomical orientations – anterior (A), posterior (P), right (R), and left (L). The image reveals findings consistent with gastrointestinal perforation, including pneumoperitoneum (free intraperitoneal air), a hallmark sign of gastrointestinal tract perforation. This visualization is critical for diagnosing and managing acute abdominal conditions.

Consultation with the endocrinology department guided the implementation of propylthiouracil (PTU; 200 mg every 8 h), intravenous hydrocortisone (100 mg), and esmolol for heart rate control. Temperature decreased to 37.8°C, and heart rate stabilized (110–120 beats/min). At 2:00 am, the patient’s abdominal symptoms worsened. A repeat CT scan revealed free intraperitoneal air, pneumatosis intestinalis, exudative gastrointestinal changes, and pelvic effusion, as illustrated in Figure 1, confirming gastrointestinal perforation. Antimicrobial therapy was escalated to sulperazon (2.0 g every 8 h). An urgent multidisciplinary consultation involving endocrinology, anesthesiology, gastrointestinal surgery, the Intensive Care Unit (ICU), and emergency medicine was convened at 5:00 am to evaluate preoperative management and perioperative risk assessment.

### 2.3. Preoperative multidisciplinary management

Endocrinology department recommendations: Preoperative optimization included increasing the dose of propylthiouracil, suppressing thyroid hormones with Lugol’s solution, controlling the heart rate with esmolol, and intensifying antimicrobial therapy to prepare the patient for surgery.

Gastrointestinal surgery recommendation: Laparoscopic repair of gastrointestinal perforation was prioritized. Nasogastric decompression, anti-inflammatory agents, acid suppression, fluid resuscitation, and broad-spectrum antimicrobials (meropenem 1.0 g every 8 h) were administered. Lugol’s solution was cautiously delivered via gastric tube (initial dose: 30 drops, adjusted incrementally every 5–8 h).

Anesthesiology assessment: Preoperative management focused on aggressively controlling the thyroid crisis to reduce circulating thyroid hormones, complemented by antimicrobial therapy and symptomatic treatment to maintain electrolyte balance. Airway evaluation and thyroid ultrasonography were completed.

ICU consultation: Heightened vigilance for cardiovascular complications was advised. Preoperative cardiac function was evaluated via B-ultrasonography. Antimicrobial prophylaxis was intensified with meropenem 1.0 g administered every 8 hours.

### 2.4. Intraoperative and postoperative management

The patient was transferred to the operating theater at 15:00 for laparoscopic gastrointestinal perforation repair. Initial vital parameters were a blood pressure of 120/80 mm Hg, a heart rate of 120 bpm, SpO₂ of 99%, a temperature of 38°C, and an oxygen flow rate of 6 L/min. Sedation was initiated with midazolam 5 mg, followed by the establishment of internal jugular venous access and left radial arterial catheterization for continuous hemodynamic monitoring. Anesthesia induction was carried out with sufentanil 25 μg, cisatracurium 16 mg, and cyclopropofol 50 mg. Tracheal intubation was uneventful, and subsequent esmolol infusion was used for heart rate modulation.

Intraoperative anesthesia maintenance involved sevoflurane inhalation combined with remifentanil and propofol infusion via a micro - pump. Emergency vasoactive agents, including epinephrine, norepinephrine, dopamine, and urapidil, were prepared for contingency use. Surgical exploration revealed copious turbid intraperitoneal purulence and a 0.7 cm antral perforation. After confirming the normal condition of abdominal viscera, thorough pus evacuation was performed, and the perforation was triply sutured with absorbable material. The peritoneal cavity was extensively irrigated with warmed saline.

By 40 minutes into the procedure, the patient showed hemodynamic stabilization: HR normalized to 90 bpm, BP equilibrated, SpO₂ reached 100%, and temperature decreased to 36 to 37°C. The total operative duration was 65 minutes, with a cumulative anesthesia time of 88 minutes. Intraoperative metrics included the administration of 1500 mL of crystalloid, an estimated blood loss of 10 mL, and a urinary output of 650 mL. Postoperative transfer to the ICU ensured continued monitoring and management.

Enteral nutrition was initiated 48 hours postoperatively via a nasogastric tube after the return of anal ventilation and the resolution of abdominal symptoms such as pain, bloating, nausea, and vomiting. A hypotonic formula was administered at an initial rate of 20 mL/h and was gradually advanced as tolerated. No signs of gastric retention were observed.

### 2.5. Postoperative outcome

The indexes of thyroid crisis were significantly improved after operation; The WBC and procalcitonin (PCT) returned to normal within 72 hours. The patient recovered and was discharged on the 10th day after surgery. At the time of discharge, outpatient gastroscopy was recommended within 1 to 2 months. The patient was also advised to consult a gastroenterologist to determine the subsequent treatment plan based on the endoscopic findings.

**Figure 2.**
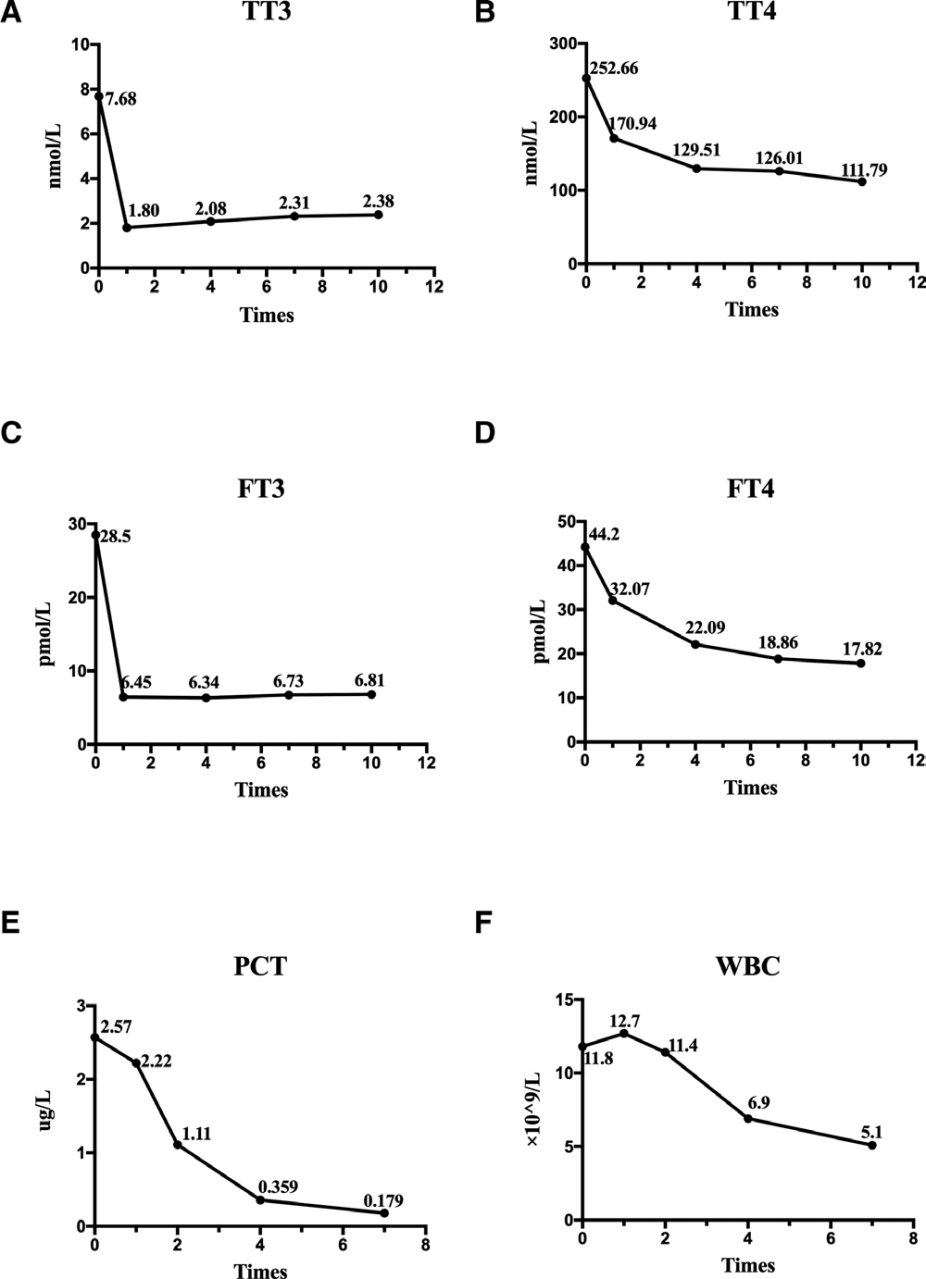
Dynamic changes of thyroid function indicators (TT3, TT4, FT3, FT4), PCT and WBC levels before and after surgery. (A–F) Figure illustrate the dynamic changes of serum total triiodothyronine (TT3, nmol/L), total thyroxine (TT4, nmol/L), free triiodothyronine (FT3, pmol/L), free thyroxine (FT4, pmol/L), procalcitonin (PCT, μg/L) and white blood cell count (WBC, ×10⁹/L) at different time points. The *x*-axis “0” represents the preoperative stage, while values ≥1 indicate postoperative days.

The trend chart of laboratory indicators of the patient’s improvement is shown in Figure 2. The progression of the patient’s disease is shown in Table 2.

**Table 2:** Timeline of patient’s clinical course.

Time	Event description	Treatment/investigation results
October 9	Onset of persistent epigastric dull pain without identifiable triggers; single episode of scant tarry stool (initially overlooked)	Self - administered omeprazole capsules (ineffective)
October 11		
Emergency admission	Progressive abdominal pain escalated to continuous cramping, necessitating ambulance transfer at 18:30	Physical exam: BP 108/53 mm Hg, HR 120/min, RR 20/min, BT 37.2°C. Imaging: Abdominal CT unremarkable. Provisional diagnosis: Acute diffuse peritonitis with concurrent hyperthyroidism
Initial management	Partial pain relief achieved	Phloroglucinol (analgesia), esomeprazole (acid suppression), ceftriaxone (antimicrobial)
20:00	Clinical deterioration: BT 39.1°C, HR 145/min. thyroid storm suspected	Thyroid function: FT3 28.58 pmol/L, FT4 44.2 pmol/L, TSH < 0.01 mIU/L. Antibodies: Elevated thyroglobulin (149.46 IU/mL) and thyroid peroxidase (>1000.00 IU/mL)
Endocrinology consultation	Thyroid storm confirmed	Treatment adjusted to: propylthiouracil 200 mg q8h, intravenous hydrocortisone 100 mg, esmolol (rate control)
October 12		
2:00 am	Acute exacerbation of abdominal symptoms	Emergency CT: Free intraperitoneal air, pneumatosis intestinalis, exudative changes, pelvic effusion (consistent with gastrointestinal perforation). Antimicrobial therapy escalated to sulperazon 2.0 g q8h
5:00 am	Multidisciplinary consultation (endocrinology, anesthesiology, general surgery, Intensive Care Unit, emergency medicine)	Preoperative optimization: propylthiouraci 200 mg q8h, Lugol’s solution (gastric tube), esomeprazole, meropenem 1.0 g q8h, esmolol 200 mg IV, hydrocortisone 100 mg, nasogastric decompression, fluid – electrolyte stabilization. Assessments: Thyroid ultrasonography, cardiac function evaluation
15:00	Laparoscopic gastrointestinal perforation repair performed	HR 90 bpm, BP stable, SpO₂100%, BT 36–37°C. The operation time was 65 min, 10 mL blood loss, 1500 mL crystalloid administered. Transferred to Intensive Care Unit for intensive monitoring
Postoperative management	Ongoing care with laboratory improvements, discharged uneventfully on day 10	Lugol’s solution titrated via gastric tube, antimicrobial therapy, acid suppression, fluid resuscitation

BP = blood pressure, BT = basal temperature, CT = computed tomography, FT3 = free triiodothyronine, FT4 = freethyroxine, HR = heart rate, TSH = thyroid-stimulating hormone.

## 3. Discussion

TS complicated with gastrointestinal perforation manifests a bidirectional pathophysiological relationship, representing a rare yet life-threatening condition. The underlying mechanisms involve thyrotoxicosis-induced hypermetabolism increasing intestinal oxygen demand, while sympathetic overactivation triggers mesenteric vasoconstriction, exacerbating intestinal hypoperfusion. This ischemic insult promotes ulcer formation and potential transmural perforation. Concurrent metabolic derangements such as hypokalemia and hyperglycemia further compromise mucosal integrity, increasing perforation risk. Gastrointestinal perforation-induced complications including intra-abdominal sepsis and systemic inflammatory response syndrome activate inflammatory cytokine release, which abnormally stimulates the hypothalamic-pituitary-thyroid axis, perpetuating thyroid hormone excess. This creates a deleterious inflammatory-endocrine storm.

Early recognition and timely intervention are therefore critical for prognosis. The diagnostic challenge lies in differentiating TS’s hypermetabolic triad (fever, tachycardia, gastrointestinal dysfunction) from perforation-related peritonitis. In our case, markedly elevated FT3/FT4 levels, suppressed TSH, and a BWPS of 65 (>45) confirmed TS, while abdominal CT localized the perforation, enabling definitive diagnosis. This precise preoperative diagnosis facilitated prompt multidisciplinary collaboration, initially focused on TS management.

Antithyroid drugs remain cornerstone therapy, including PTU and methimazole (MMI). Our patient received PTU 200 mg via nasogastric tube every 8 hours, selected for its inhibition of peripheral T4-to-T3 conversion to mitigate hypermetabolic effects. The Japanese Endocrine Society nationwide survey demonstrated comparable outcomes between MMI and PTU in disease severity and mortality.^[8]^ However, nasogastric administration of antithyroid drugs in bowel obstruction remains controversial. β-blockers are essential for countering sympathetic hyperactivity. While nonselective agents like propranolol predominate in China, reports associate them with circulatory collapse in cardiac-compromised patients.^[9,10]^ Consistent with Japanese Thyroid Association guidelines,^[1]^ we employed esmolol (200 mg continuous IV infusion) as the selective β1-blocker of choice. Hydrocortisone (100 mg IV every 8 h) provided triple benefits: thyroid hormone release suppression, T4-to-T3 conversion inhibition, and adrenal crisis prevention.

Regarding surgical timing, while the 2024 guidelines from the Society of American Gastrointestinal and Endoscopic Surgeons recommend immediate surgical repair within 6 to 12 hours following gastrointestinal perforation, uncontrolled TS significantly elevates perioperative mortality due to hemodynamic instability. Therefore, a comprehensive individualized risk-benefit assessment becomes imperative. In our case, after achieving controlled intra-abdominal infection through effective measures and complete or partial resolution of thyrotoxicosis-related hypermetabolic symptoms with the aforementioned medical therapies, surgery was deliberately delayed beyond the 12-hour window. Despite this delay, the patient maintained stable perioperative hemodynamics and achieved favorable outcomes. This stands in stark contrast to prior reports advocating emergent surgery despite uncontrolled thyrotoxicosis.^[11]^

Laparoscopic surgery is currently widely employed in various surgical procedures and offers distinct clinical advantages over traditional laparotomy, including reduced invasiveness, decreased postoperative pain, and shorter hospitalization.^[12]^ However, pneumoperitoneum frequently induces intraoperative hemodynamic alterations, rendering its feasibility controversial in TS-associated hemodynamic instability. In this case, laparoscopic surgery was selected. Through a combined protocol of restricted insufflation pressure (<10 mm Hg), abbreviated operative duration (65 min), continuous monitoring of central venous pressure and lactate levels, avoidance of adrenergic agonists, and prioritized use of cardiovascular-sparing anesthetic agents (e.g., sevoflurane and remifentanil for maintenance), the patient maintained hemodynamic stability throughout the procedure, enabling rapid postoperative extubation.

Postoperatively, rigorous monitoring was mandated to address the combined cardiopulmonary impacts of sepsis and incompletely controlled TS. Targeted antibiotic therapy guided by peritoneal bacterial culture results was implemented to minimize antibiotic resistance. Additionally, hypotonic enteral nutrition (20 mL/h) was initiated at 24 hours postoperatively to preserve intestinal mucosal integrity and reduce bacterial translocation. Notably, the patient exhibited a >50% decline in PCT within 48 hours without delayed gastric emptying. All postoperative parameters demonstrated favorable recovery, culminating in uneventful discharge on postoperative day 10.

Study limitations: This is a single-center case report with limited external validity. The lack of long-term follow-up prevents evaluation of recurrence risk or thyroid function stability. Larger studies are needed to guide perioperative and endocrinologic management in similar scenarios.

## 4. Conclusion

This case offers 3 clinical insights: Suspect TS in perforation patients with unexplained tachycardia, fever, or hyperthyroidism history; consider deliberate surgical delay when infection is controlled to optimize TS management; laparoscopic surgery combined with TS-tailored anesthesia may enhance outcomes in high-risk populations.

## Author contributions

**Data curation:** Ling Ye.

**Project administration:** Weiqiao Zhang.

**Writing – original draft:** Ling Ye.

**Writing – review & editing:** Weiqiao Zhang.
